# The mediating effect of community identity and the moderating effect of social comparison in the relationship between residential mobility and sense of meaning in life

**DOI:** 10.3389/fpsyg.2025.1501060

**Published:** 2025-03-05

**Authors:** Song Weifang, Duan Majie, Zhao Na

**Affiliations:** ^1^Department of Police General Education, Zhengzhou Police College, Zhengzhou, Henan, China; ^2^School of Sociology and Psychology, Central University of Finance and Economics, Beijing, China

**Keywords:** residential mobility, sense of meaning in life, presence of meaning, search for meaning, community identity, social comparison

## Abstract

**Introduction:**

Numerous studies have documented the adverse effects of residential mobility; however, its relationship with the sense of meaning in life remains underexplored. This study examines the mechanisms by which residential mobility influences the subjective sense of meaning in life, focusing on the mediating role of community identity and the moderating role of social comparison.

**Methods:**

We used the platform “Creator of Data and Model” to conduct an online survey. The sample of adult participants recruited were aged <35 years (85.0%).

**Results:**

The results revealed that residential mobility negatively predicts a sense of meaning in life, particularly through reduced community identity. Social comparison moderated these effects, with high social comparison tendencies exacerbating negative outcomes in specific dimensions.

**Discussion:**

These findings advance our understanding of the psychological consequences of residential mobility and provide practical insights into supporting the well-being of mobile populations.

**Conclusion:**

Enhancing community identity can mitigate the adverse effects of mobility, whereas tailored interventions for socially comparable individuals may improve their well-being.

## 1 Introduction

Residential mobility, a common phenomenon in contemporary societies, refers to the frequency with which individuals relocate to different residences. This behavior is typically driven by the pursuit of economic opportunities, improved living conditions, or career advancement (Greenwood, 2019; Rabe and Taylor, 2010). While residential mobility is generally viewed as an adaptive response to these factors, its psychological effects—particularly concerning individuals’ sense of meaning in life—remain underexplored (Dai and Li, 2018; Zhang et al., 2021). Existing research has demonstrated that residential mobility can disrupt family and social connections and lead to a decline in psychological stability and overall well-being (Choi and Oishi, 2020; Henkens et al., 2024; Li et al., 2019; Oishi and Schimmack, 2010). Additionally, mobility can significantly impact individuals’ self-concept (Oishi et al., 2007; Zhang et al., 2020) and their willingness to explore new social environments (He et al., 2023). These factors are intrinsically connected to an individual’s sense of meaning in life (Wang et al., 2023). In this study, we explore how residential mobility influences life meaning, with a particular focus on the mediating role of community identity and the moderating role of social comparison.

### 1.1 Meaning in life and residential mobility

Meaning in life refers to an individual’s understanding of and concern for the factors that give significance to their existence. This multifaceted concept is generally divided into two primary dimensions: the presence of meaning and the search for meaning (King and Hicks, 2021; Steger et al., 2006). The presence of meaning pertains to the degree to which individuals perceive their lives as meaningful and fulfilling, while the search for meaning involves the ongoing pursuit of purpose and understanding (Steger et al., 2006). This carried unique psychological and emotional implications.

The presence of meaning is positively correlated with subjective well-being, life satisfaction, and mental health (Baumeister, 1991; Steger et al., 2006). Individuals who perceive their lives as meaningful are more likely to report higher levels of psychological well-being, including greater life satisfaction, enhanced coping strategies, and lower levels of stress and depression (Steger et al., 2006; Kleftaras and Psarra, 2012; Zhao et al., 2017). A strong sense of meaning in life has consistently been linked to positive mental health outcomes, including reduced anxiety and depressive symptoms (Li et al., 2023; Zhao et al., 2017). In contrast, the search for meaning may be associated with negative emotional outcomes, such as existential anxiety or depression, especially when individuals experience disconnection or a lack of purpose (Heine et al., 2006; Park, 2010; Steger et al., 2009). These two dimensions are interrelated yet distinct, and previous research often treated them as a unified construct when studying life meaning. Therefore, in this study, we initially examined life meaning as a whole, providing a basis for the analysis, before separately exploring the two dimensions.

Residential mobility, or the frequency of relocation, is known to disrupt individuals’ sense of continuity and stability, which are essential for maintaining a coherent self-concept and sense of purpose (Oishi et al., 2007). Frequent relocations challenge individuals’ ability to form and sustain lasting relationships and stable environments, both of which are crucial for psychological well-being and the development of a meaningful life narrative (Gillath and Keefer, 2016). Research has shown that the disruption of these aspects, particularly social ties and personal stability, can directly diminish an individual’s sense of meaning in life. For instance, individuals who move frequently may struggle with a lack of social support, weakened community ties, and a diminished sense of belonging—all of which contribute to a reduced sense of life purpose (Diener and Tay, 2015; Hagge and Schacht, 2024).

The direct effect of residential mobility on life meaning can be understood through the lens of continuity theory, which posits that individuals derive meaning from the continuity of their relationships, self-concept, and life experiences (Atchley, 1989). According to Atchley (1989), continuity theory suggests that individuals maintain a sense of identity and purpose by preserving consistent patterns of behavior and relationships across different life stages. Frequent relocations disrupt this continuity, leading to a fragmented sense of self and a diminished ability to construct meaningful life narratives. This theory aligns with findings suggesting that frequent relocations exacerbate feelings of alienation and dissatisfaction, as individuals continuously adjust to new environments without the stability of established social networks (Liu et al., 2017; Park, 2010).

Based on this, the first hypothesis of this study is proposed: “Residential mobility negatively predicts the sense of meaning in life. Higher levels of residential mobility are expected to be associated with lower levels of meaning in life, which will manifest across both the presence of meaning and the search for meaning dimensions.”

### 1.2 The role of community identity

While residential mobility directly impacts meaning in life, community identity plays a significant mediating role in this relationship. Community identity, which refers to the emotional and functional attachment individuals have to a social environment, is crucial for maintaining a sense of belonging and stability (Heine et al., 2006). Emotional identity refers to the deep emotional connections individuals form with their community, fostering a sense of belonging and psychological comfort (McMillan and Chavis, 1986). Conversely, functional identity includes the practical benefits provided by the community, such as resources, safety, and social support—elements that are necessary for coping with life’s challenges (Carter et al., 2023). Both dimensions are integral to shaping an individual’s sense of meaning in life, as they provide the emotional security and stability needed to construct and sustain coherent life narratives (Haslam et al., 2005; Hoog and Pat-El, 2024).

Research has shown that disruptions in community identity caused by frequent relocations can diminish individuals’ sense of meaning in life. When individuals cannot form strong emotional connections with a community or access the resources and support offered by a stable community, they might struggle to maintain a coherent life narrative (Oishi, 2010b). This disruption limits access to the social and emotional resources necessary for constructing a meaningful life, leading to an increased sense of alienation and a decreased sense of life purpose. Therefore, community identity serves as a crucial mediator in the relationship between residential mobility and life meaning, where the loss of community identity exacerbates the negative effects of residential mobility on meaning in life.

Based on the above discussion, the second hypothesis is formulated as follows: “Community identity mediates the relationship between residential mobility and the sense of meaning in life. Frequent residential moves are expected to lead to a loss of community identity, which in turn diminishes the sense of meaning in life, including both the presence of meaning and the search for meaning.

### 1.3 The role of social comparison

Social comparison theory, initially proposed by Festinger (1954), suggests that individuals evaluate their attributes, abilities, and opinions by comparing themselves to others. This tendency plays a crucial role in shaping emotional well-being and self-concept, particularly in dynamic and uncertain environments (Buunk and Gibbons, 2007). Social comparison can either serve as a source of motivation and self-improvement or lead to negative emotional outcomes, such as anxiety, low self-esteem, and depression (Steers et al., 2014; Vogel et al., 2015; Wu et al., 2019). In the context of residential mobility, social comparison may exacerbate the psychological challenges associated with frequent relocations. Research indicates that individuals who are more prone to social comparison often experience heightened negative emotions, such as dissatisfaction and anxiety when adjusting to new environments (Buunk and Gibbons, 2007). These individuals may be particularly vulnerable to feelings of inadequacy when comparing themselves to others who appear more successful or integrated into their new environment, reinforcing their sense of instability and dissatisfaction (Steers et al., 2014).

Conversely, individuals with relatively low tendencies for social comparison may be better equipped to handle the uncertainty and instability associated with mobility, potentially mitigating the negative effects on their sense of meaning in life. Studies show that individuals who engage less in social comparison tend to experience better psychological adjustment during life transitions, such as relocation, by focusing more on personal growth and less on external benchmarks (Kashdan and Ciarrochi, 2013; Steers et al., 2014). This ability to regulate emotions through reduced social comparison can facilitate a smoother transition in unstable environments. Individuals with a tendency to engage in social comparisons may also be more prone to maladaptive coping mechanisms, such as ruminating over social comparisons or feeling inadequate when faced with a perceived lack of social success (Vogel et al., 2015). This ruminative tendency exacerbates emotional distress, particularly in situations where individuals feel their current social environment does not align with their expectations or self-concept (Hu and Guo, 2021; Zhang et al., 2023). This supports the argument that a lower tendency for social comparison can enable a healthier psychological adjustment to the challenges posed by residential mobility, protecting one’s sense of meaning in life from the disruptive effects of frequent relocation.

Based on this, the third hypothesis is proposed: “Social comparison moderates the relationship between residential mobility and the sense of meaning in life. Individuals with higher tendencies for social comparison will experience greater disruptions in their sense of meaning (in terms of both presence and search for meaning) because of residential mobility.”

## 2 Materials and methods

### 2.1 Participants

In this study, we employed an online survey method using the platform “Creator of Data and Model”; 533 adult participants were recruited using convenience sampling, with a response rate of 93.81%. After excluding 33 participants whose response times were either too short (less than 300 s) or excessively long (greater than 1,800 s), the final sample consisted of 500 individuals. All the participants provided informed consent before participating in the study.

The sample included adults aged <35 years (85.0%). Participants represented a diverse range of socioeconomic backgrounds and geographical regions across China. Concerning educational attainment, 12.8% of participants had completed junior college or lower, 73.8% held a bachelor’s degree, and 13.4% had obtained a master’s degree or higher. Regarding occupation, 29.6% were adult students, 16.8% were public sector employees or civil servants, 47.8% were corporate employees, and the remaining 5.8% of participants belonged to other occupational categories.

### 2.2 Measurements

#### 2.2.1 Residential mobility

Residential mobility was assessed by asking participants to report the cumulative number of times they had changed their place of residence since birth. Responses were recorded numerically (e.g., “1” for one move, “2” for two moves) following the methodology established by Oishi et al. (2007).

#### 2.2.2 Community identity

Community identity was measured using an 8-item scale developed by Xin and Ling (2015) that captured two dimensions: functional and emotional identity. The items were rated on a 6-point Likert scale. The scale demonstrated excellent reliability (*Cronbach’s alpha* = 0.83).

#### 2.2.3 Meaning in life

The Meaning in Life Questionnaire (MLQ; Steger et al., 2006) was adapted to the Chinese context (Wang and Dai, 2008). The questionnaire (*Cronbach’s alpha* = 0.77) consists of 10 items divided into two subscales: the ‘Presence of Meaning’ (*Cronbach’s alpha* = 0.90) and the ‘Search for Meaning’ (*Cronbach’s alpha* = 0.91).

#### 2.2.4 Social comparison

The Social Comparison Orientation Scale (Onyekachi et al., 2024) was used to evaluate participants’ tendencies to compare themselves with others. This scale also demonstrated high internal consistency in this study (*Cronbach’s alpha* = 0.88).

### 2.3 Statistical analyses

Data analysis was performed using SPSS software (version 23.0). Descriptive statistics and correlations among the key study variables were first examined. The Hayes’ PROCESS macro (version 4.1) was used to test the proposed hypotheses. Specifically, Model 4 was used to examine the mediating role of community identity, and Model 1 was used to test the moderating role of social comparison. All demographic variables, including gender, age, education, and occupation, were controlled for in the analyses to ensure the robustness of the results.

### 2.4 Common method bias test

To address the potential common method bias due to self-reported measures, a single-factor test was conducted. Seven factors with eigenvalues greater than 1 were extracted, with the largest factor accounting for only 23.04% of the variance, which is well below the threshold of 40%. This indicates that the common method bias did not significantly affect the study results.

## 3 Results

Table 1 presents the descriptive statistics and correlations between the study variables. The results showed a significant negative relationship between residential mobility and both community identity (*r* = −0.22, *p* < 0.01) and overall sense of meaning in life (*r* = −0.11, *p* < 0.05). Community identity was positively associated with the overall sense of meaning in life (*r* = 0.38, *p* < 0.01). Interestingly, residential mobility and community identity did not exhibit a significant relationship with the ‘Search for Meaning’ subscale.

**Table 1:** Descriptive statistics and correlations for all study variables (*n* = 500).

	*M*	*SD*	1	2	3	4	5	6	7	8	9	10
1.Sex	1.66	0.48	1									
2.Age	1.78	0.82	−1.22^**^	1								
3.Education level	2.01	0.51	0.03	−0.07	1							
4.Occupation	2.41	1.11	−1.01^*^	0.47^**^	−0.16^**^	1						
5.Residential mobility	3.18	1.32	0.11^*^	0.21^**^	−0.03	0.23^**^	1					
6.Community identity	36.55	5.64	−0.19^*^	0.18^**^	−0.01	−0.22^**^	−0.22^**^	1				
7.Meaning in life	52.26	8.02	−0.14^**^	0.12^**^	0.10^*^	−0.11^**^	−0.11^*^	0.38^**^	1			
8.Presence of meaning	26.10	6.05	−0.23^**^	0.29^**^	0.06	−0.14^**^	−0.14^**^	0.50^**^	0.69^**^	1		
9.Search for meaning	26.16	5.86	0.05	0.13^**^	0.08	−0.01	−0.01	0.01	0.66^**^	−0.09^*^	1	
10.Social comparison	45.07	8.81	−0.08	−0.04	0.05	−0.06	−0.01	0.18^**^	0.33	0.08	0.37^**^	1

^*^p < 0.05, ^**^p < 0.01, ^***^p < 0.001.

In addition to the primary variables, demographic factors such as gender, age, educational level, and occupation were found to influence the sense of meaning in life. These variables were controlled for in subsequent analyses to isolate the effects of residential mobility and community identity.

### 3.1 The mediating role of community identity

**Figure 1:**
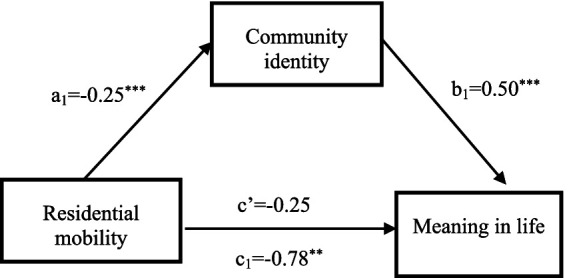
The mediating role of community identity between residential mobility and meaning in life. **p < 0.01, ***p < 0.001.

To scrutinize the intermediary function of community identity in the nexus between residential mobility and the overall sense of meaning in life, we employed Process (Version 4.1) Model 4. After controlling for pertinent demographic covariates, our results reveal that residential mobility exerts a marked negative influence on both the sense of meaning in life (*β* = −0.78, *t* = −2.81, *p* = 0.005, 95% *CI*: [−1.33, −0.23]) and community identity (*β* = −1.06, *t* = −5.61, *p* < 0.001, 95% *CI*: [−1.43, −0.69]). When both residential mobility and community identity were simultaneously considered in predicting the sense of meaning in life, community identity emerged as a significant positive predictor (*β* = 0.50, *t* = 7.98, *p* < 0.001, 95% *CI*: [0.38, 0.62]), whereas the direct negative effect of residential mobility on the sense of meaning in life diminished to non-significance (*β* = −0.25, *t* = −0.93, *p* = 0.35, 95% *CI*: [−0.78, 0.28]). This outcome underscores the mediating role of community identity, thereby supporting the first and second hypotheses (Figure 1).

**Figure 2:**
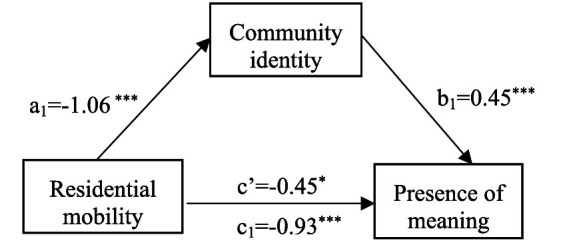
The mediating role of community identity between residential mobility and presence of meaning. *p < 0.05, ***p < 0.001.

Previous research has demonstrated that the two dimensions—the ‘Presence of Meaning’ and ‘Search for Meaning’—are distinct constructs with different psychological implications (Steger et al., 2006). The presence of meaning refers to the degree to which individuals feel their lives are purposeful and fulfilled, whereas the search for meaning reflects an ongoing quest for purpose. These dimensions often display different relationships with psychological outcomes such as well-being, depression, and life satisfaction, and may therefore not be appropriately captured by the total score (Steger et al., 2009). Accordingly, we conducted further research on the sense of meaning in life dimensionally, separating the ‘Presence of Meaning’ and ‘Search for Meaning’ subscales of the MLQ. Considering the absence of a correlation between residential mobility and the search for meaning, the mediation analysis focused solely on the presence of the meaning dimension. Upon controlling for demographic confounders, residential mobility was found to significantly predict the presence of meaning (*β* = −0.93, *t* = −4.73, *p* < 0.001, 95% *CI*: [−1.32, −0.55]) and community identity (*β* = −1.06, *t* = −5.61, *p* < 0.001, 95% *CI*: [−1.44, −0.69]). When both variables concurrently predicted the presence of meaning, community identity emerged as a robust positive predictor (*β* = 0.45, *t* = 10.79, *p* < 0.001, 95% *CI*: [0.37, 0.54]), whereas residential mobility retained a significant negative effect, albeit attenuated (*β* = −0.45, *t* = −2.46, *p* = 0.01, 95% *CI*: [−0.81, −0.09]). This pattern suggests partial mediation by community identity, with a mediation effect size of −0.48 and a 95% bootstrap confidence interval (CI) of [−0.69, −0.30], accounting for 51.79% of the total effect (Figure 2).

### 3.2 The moderating effect of social comparison

Using Model 1 within the PROCESS framework, we empirically examined the moderating influence of social comparison. The findings revealed a non-significant moderating effect, as evidenced by the interaction regression coefficient between residential mobility and social comparison, which yielded *β* = −0.01, *t* = −0.22, *p* = 0.82, with a 95% CI spanning zero ([−0.06, 0.05]). This result indicates that social comparison does not significantly alter the relationship between residential mobility and the overall sense of meaning in life.

To further explore the nuanced role of social comparison, its moderating effects were subsequently analyzed separately across two distinct dimensions of meaning in life: the ‘Presence of Meaning’ and ‘Search for Meaning.’

**Figure 3:**
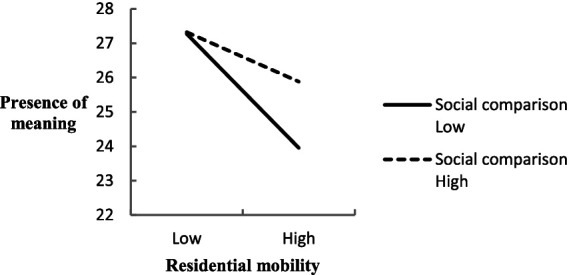
The moderating effect of social comparison between residential mobility and presence of meaning.

Regarding the ‘Presence of Meaning’ dimension, the analysis yielded a significant moderating effect, with an interaction regression coefficient of *β* = 0.04, *t* = 2.014, *p* = 0.04, and a CI excluding zero ([0.00, 0.09]). This suggested that social comparison positively moderates the relationship between mobility and the experience of meaning, indicating that individuals prone to social comparison exhibit significantly higher levels of perceived meaning at similar levels of residential mobility than those less inclined toward such comparisons (Figure 3).

**Figure 4:**
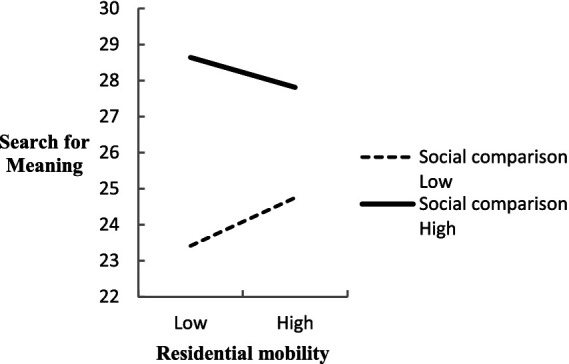
The moderating effect of social comparison between residential mobility and search for meaning.

Conversely, in the context of the search for the meaning dimension, the analysis revealed a significant negative moderating effect, characterized by an interaction regression coefficient of *β* = −0.05, *t* = −2.28, *p* = 0.02, with a CI excluding zero ([−0.09, −0.01]). This underscored the fact that social comparison negatively influences the relationship between mobility and the active pursuit of meaning, as individuals with a lower tendency toward social comparison exhibit a more pronounced search for meaning as their residential mobility increases, whereas those highly prone to social comparison demonstrate a diminished search for meaning under similar circumstances (Figure 4).

In summary, social comparison exerts a dual influence on the two dimensions of meaning in life, positively moderating the relationship between mobility and the presence of meaning, and negatively moderating the relationship between mobility and the search for meaning. Consequently, the third hypothesis was partially supported.

## 4 Discussion

This study revealed a significant negative correlation between residential mobility and sense of meaning in life, specifically in the ‘Presence of Meaning’ dimension, while showing no direct impact on the search for meaning. These findings align with those of prior research (Oishi and Tsang, 2022), highlighting how frequent relocation disrupts psychological continuity and weakens individuals’ connections with their social environments, thereby reducing opportunities for self-expansion (Zhang and Li, 2018). Consequently, individuals often experience feelings of loneliness, confusion, and diminished social identity (Shi et al., 2022). These disruptions erode a sense of belonging (Shi et al., 2021), ultimately undermining the perceptions of meaning in life. This underscores the distinctive role of the presence of meaning as a critical component of the overall meaning in life.

In contrast, the absence of a relationship between residential mobility and the search for meaning highlights the independence of these two constructs. The search for meaning is influenced by various moderating factors, such as individual motivation and existing perceptions of purpose (Zhang and Li, 2018). This finding indicates that although residential mobility disrupts the stability required for a coherent sense of presence, it does not necessarily reduce individuals’ inclination to pursue meaning in life. These results underscore the nuanced relationship between the two dimensions of meaning in life, supporting the need for separate analyses in future research.

Many studies have focused on a single dimension of meaning in life due to its complex association with well-being. For example, the ‘Presence of Meaning’ dimension is closely linked to greater life satisfaction and subjective well-being, whereas the ‘Search for Meaning’ dimension may correlate with stress or existential anxiety under certain conditions (King and Hicks, 2021; Steger et al., 2006). Differentiating these dimensions is essential, as they often exhibit distinct relationships with psychological and environmental factors. By analyzing these dimensions independently, we aim to clarify the dual impact of residential mobility on meaning in life. Specifically, the presence of meaning reflects stability and coherence supported by a strong community identity, while the search for meaning is shaped by broader motivational and contextual factors, such as tendencies toward social comparison. This theoretical distinction, outlined by Steger et al. (2006), aligns with prior empirical findings (Hu and Wang 2023; Lavigne et al., 2012) that suggest combining these dimensions into a single construct can obscure the underlying psychological dynamics. By separating the two, this study enhances the clarity and relevance of its findings, providing practical insights for addressing the challenges faced by mobile populations.

Moreover, this study identified community identity as a crucial mediator in the relationship between residential mobility and the presence of meaning. Extended residential stability enhances belonging and identity, which are foundational for experiencing a meaningful life (Xin and Ling, 2015). In contrast, frequent relocation often leads to rootlessness, social isolation, and difficulty forming meaningful connections (Dou et al., 2019). These findings emphasize the importance of fostering community identity to mitigate the psychological challenges of mobility. Practical interventions could include community-building initiatives aimed at enhancing individuals’ sense of belonging, which may be particularly valuable in contexts of frequent residential mobility, such as urban areas with high rates of migration. Notably, this mediation effect is specific to the presence of meaning, as neither residential mobility nor community identity significantly relates to the search for meaning.

Additionally, our study revealed that the total scale score for social comparison did not exhibit significant moderating effects. This likely reflects the multifaceted nature of social comparison, which influences the two dimensions of meaning in life in opposing ways. Dimensional analyses showed that social comparison positively moderates the relationship between residential mobility and the presence of meaning, while negatively moderating the relationship with the search for meaning. This finding highlights the need to treat the presence of meaning and search for meaning as distinct constructs.

In this study, the presence of meaning reflects stability and coherence reinforced by a strong community identity, which social comparison can positively enhance when individuals perceive their lives as fulfilling. Conversely, the search for meaning is influenced by broader contextual and motivational factors, including excessive social comparison, which may increase dissatisfaction and confusion, hindering the pursuit of meaning. These opposing effects help explain why the total score for social comparison obscures its nuanced role in moderating the relationship between residential mobility and the two dimensions of meaning in life. This approach aligns with previous research (Lavigne et al., 2012; Steger et al., 2006) that demonstrated the value of independent dimensional analyses in uncovering unique psychological processes. By examining these dimensions separately, this study highlights the dual role of social comparison and its context-dependent influence on psychological outcomes. These findings deepen our understanding of how individual differences in social comparison tendencies interact with environmental and psychological factors to shape experiences of meaning in life.

Finally, this study offers a potential explanation for the modest negative correlation observed between the presence of meaning and the search for meaning in life (*r* = −0.094). This finding aligns with research conducted in the United States (Steger et al., 2008) but contrasts with the positive correlation reported in Chinese studies (Wang and Dai, 2008). Two key factors may explain this discrepancy. First, the data were collected after the COVID-19 pandemic, a period marked by a heightened awareness of life’s fragility, prompting individuals to prioritize immediate meaning over the pursuit of long-term, undefined goals. Second, Wang and Dai (2008) attributed their findings to cultural differences such as the contrast between American individualism and Chinese collectivist interdependence. However, cultural disparities may diminish as societies become more interconnected and multicultural integration deepens. This shift is reflected in the weak negative correlation observed in this study, which deviates not only from earlier Chinese research but also from the moderate negative correlations observed in some contexts in the United States. This finding suggests that the relationship between the two dimensions of meaning in life is dynamic and shaped by cultural, temporal, and contextual factors.

While many people choose to migrate in search of better job opportunities, higher income, and career advancement, these economic and career factors undoubtedly play a leading role in mobility decisions. However, our results show that frequent relocation often negatively impacts individuals’ sense of meaning in life, especially concerning the psychological needs and personality differences of mobile populations. This study advances existing research by examining the psychological mechanisms linking residential mobility and a sense of meaning in life, with a particular focus on the mediating role of community identity and the moderating role of social comparison. These findings provide valuable insights into the dual impact of residential mobility on psychological well-being and elucidate both its detrimental effects and the conditions under which these effects may be mitigated. The practical implications emphasize the need for interventions aimed at fostering community identity to enhance the well-being of mobile populations.

Although this study offers valuable insights, it has several limitations that warrant further discussion. First, it assumes that residential mobility in modern society is driven primarily by aspirations for future development or an improved quality of life, implying an upward mobility trajectory. Consequently, the analysis focused solely on the frequency of mobility, without differentiating between upward and downward mobility. This oversight limits our ability to capture the nuanced psychological and social implications of different mobility types. Future research should aim to classify residential mobility in detail, examining how various types (e.g., upward vs. downward mobility) uniquely influence the sense of meaning in life and the associated psychological mechanisms.

Second, the observed correlation between the two dimensions of meaning in life—the ‘Presence of Meaning’ and ‘Search for Meaning’—deviates from previous findings. This study found a modest negative correlation, which contrasts with the positive correlation observed in previous studies conducted in China. Although cultural and temporal explanations have been proposed, these findings require further verification. Additionally, as cultural contexts and societal dynamics continue to evolve, future studies should explore how these changes differentially affect the interplay between these dimensions in diverse populations.

Third, this study adopted a predominantly unidirectional approach, examining how residential mobility influences the sense of meaning in life and community identity. However, it is equally plausible that the relationship between community identity (mediator) and the sense of meaning in life (dependent variable) could operate in the opposite direction, where changes in the sense of meaning in life serve as a mediator between residential mobility and community identity. For instance, frequent residential mobility might first disrupt an individual’s sense of meaning in life, which subsequently undermines their community identity. To test this possibility, future research should employ longitudinal or experimental designs to explore if the sense of meaning in life acts as a mediator in this causal chain. Such investigations could provide a more nuanced understanding of how residential mobility impacts individual well-being and community integration. Additionally, future studies should address the temporal ordering of these variables, as this would clarify the directional nature of their relationships and enhance the robustness of the findings.

Finally, although these findings highlight the moderating role of social comparison, this study did not explore other individual differences or contextual factors that may influence these relationships. For instance, personality traits such as openness to experience, resilience, and extraversion can shape how individuals respond to frequent relocations. Similarly, broader societal factors, including urbanization trends and economic stability, may interact with mobility and affect psychological outcomes. Future research should incorporate these additional variables to develop a more holistic framework for understanding the psychological consequences of residential mobility.

In summary, while this study advances our understanding of the relationship between residential mobility and a sense of meaning in life, it also highlights critical areas for further exploration. Addressing these limitations through more nuanced and comprehensive methodologies will not only refine the theoretical framework but also offer practical insights for supporting mobile populations in increasingly dynamic societies.

## Data Availability

The original contributions presented in the study are included in the article/Supplementary material, further inquiries can be directed to the corresponding author.
